# An evaluation of the impact of abattoir processing on the levels of *Campylobacter* spp. and *Enterobacteriaceae* on broiler carcasses

**DOI:** 10.3389/fmicb.2025.1613058

**Published:** 2025-07-16

**Authors:** Rita Papoula-Pereira, Khalid Abdulla, Georgia Silver, Abigail Kellett, Dragan Antic

**Affiliations:** Department of Livestock and One Health, Institute of Infection, Veterinary and Ecological Sciences, University of Liverpool, Neston, United Kingdom

**Keywords:** *Campylobacter*, *Enterobacteriaceae*, broiler carcass, abattoir, interventions, ultrasound-hot water, process hygiene criteria, antibiotic resistance

## Abstract

**Background:**

Twenty years since the monitoring of foodborne diseases started in the EU and United Kingdom, *Campylobacter* infection is still the most reported zoonosis. One of the crucial reasons for this is thought to be an increase in *Campylobacter* virulent strains in the chicken meat as a consequence of insufficient and/or inadequate controls on farm and during chicken slaughter and processing. This study aimed to investigate the impact of abattoir processing on the levels of *Campylobacter* spp. and *Enterobacteriaceae* on broiler carcasses, including the effect of hot water carcass immersion and ultrasound intervention, the abattoir’s compliance with process hygiene criteria (PHC) and antimicrobial resistance in *Campylobacter* spp. strains.

**Methods:**

Neck skin samples (*n* = 270) were taken from seven broiler batches over seven sampling days in one abattoir, immediately after defeathering, evisceration, hot water immersion/ultrasound intervention and air-chilling (40 samples per day/batch). Quantification of *Campylobacter* spp. and *Enterobacteriaceae* was performed based on ISO methods following *Campylobacter* spp. confirmation on the MALDI-TOFF and PCR. Antimicrobial susceptibility testing of *Campylobacter* spp. was performed via disc diffusion method using EUCAST guidelines.

**Results:**

*Campylobacter jejuni* was confirmed in 93.7%, *C. coli* in 1.1% and *Campylobacter* spp. in 1.9% of samples. Abattoir processing significantly reduced final carcass microbial load, with an overall reduction in *Campylobacter* and *Enterobacteriaceae* levels of 1.14 log_10_ and 1.43 log_10_, respectively. Hot water immersion and ultrasound intervention substantially decreased *Campylobacter* levels by 0.85 log_10_ and *Enterobacteriaceae* levels by 0.82 log_10_. The abattoir was found unsatisfactory regarding compliance with PHC for *Campylobacter* levels within the sampling window, but satisfactory when the new proposed PHC for *Enterobacteriaceae* levels was applied. Antimicrobial resistance was found in *Campylobacter* isolates from all seven chicken batches, and 48.7% of isolates showed resistance to at least one antibiotic. Most isolates exhibited resistance to tetracycline (45%), nalidixic acid (41%), and ciprofloxacin (39%). Multidrug resistance was found in 2.7% of *Campylobacter* isolates, with combined resistance to ciprofloxacin, erythromycin and tetracycline in 1.6% of isolates.

**Conclusion:**

This study confirmed significant reduction of microbial load on chicken carcasses during abattoir processing, with an emphasis on the importance of using interventions in meat industry. The prevalence of resistance to ciprofloxacin and tetracycline is not declining in *Campylobacter* spp. on chicken meat, despite antimicrobial stewardship initiatives, and the presence of multidrug resistant strains may be of public health concern.

## Introduction

Over the past 20 years, *Campylobacter* infection have been the most commonly reported zoonosis in EU member states (EFSA and ECDC, 2024) and in the United Kingdom (ACMSF, 2019; UKHSA, 2024). European Food Safety Authority (EFSA) estimated that there were 9 million cases of campylobacteriosis annually in the EU leading to the disease burden of 0.35 million disability-adjusted life years (EFSA, 2011). In the United Kingdom, the estimates are that *Campylobacter* kills 100 people each year, with case numbers rising since 2005 and annual economic burden being around £900 million (out of a total of around £1.5 billion for all foodborne infections) (FSA, 2013). Most human cases are mild and self-limiting, but in the immunosuppressed, pathogenesis can be further complicated and patients may require treatment i.e., due to Guillan-Barré Syndrome (Igwaran and Okoh, 2019; WHO, 2015) or potentially even die (Holland et al., 2020). The impacts of this infection on quality of life, healthcare and productivity can be therefore substantial.

Approximately 90% of all *Campylobacter* cases are believed to be caused by *C. jejuni*, and to lesser extent by *C. coli* (Liu et al., 2022). Source attribution studies have identified chicken meat as the most frequent cause of *Campylobacter* infections (Cody et al., 2019). In the UK the estimate is that about 70% of *C. jejuni* and just under 50% of *C. coli* human infections have been linked to the chicken source (McCarthy et al., 2021). Whilst most cases of human infection with *Campylobacter* are treatable, there is a rising concern of antimicrobial resistance, with the WHO ranking fluoroquinolone-resistant *Campylobacter* as “priority pathogens” (WHO, 2017). Because of this, there has been a change in choice of treatment with macrolides taking preference over the historic use of quinolones (Bolinger and Kathariou, 2017). In Europe, it is mandatory to monitor antimicrobial resistance in food producing animals, food and humans (ECDC, 2016).

It is very well known that the initial carcass contamination occurs during abattoir processing and the prevalence of *Campylobacter* on carcasses is highest immediately post abattoir processing (Buncic et al., 2017). This is not surprising, since *C. jejuni* load in caeca can reach 10^9^ CFU/g (Hermans et al., 2011; Wagle et al., 2020) and gut spillage during defeathering and evisceration process can be a very frequent event (Buncic et al., 2017). To control *Campylobacter* at slaughter, Process Hygiene Criteria (PHC) at abattoir level have been used since 2018. Weekly testing of 5 samples (each sample made of 3 neck skins) is performed over a 10-week period, and no more than 15/50 samples (or 30%) is allowed to exceed 1,000 CFU/g (3 log_10_) for satisfactory result (from 1st January 2025 no more than 10/50 samples (or 20%) (EC, 2017). The results indicate or verify whether the food business operators’ (FBO) production processes and their Hazard Analysis and Critical Control Points (HACCP) plan are effectively complying with the regulatory framework (Cegar et al., 2022). Furthermore, these results based on the testing for indicator bacteria can be used to compare the performance between abattoirs for the purpose of their risk categorization (Cegar et al., 2022; Salines et al., 2023). Beside *Campylobacter*, testing for *E. coli* and *Enterobacteriaceae*, common commensal indicator microorganisms, has been proposed for the purpose of PHC (Cegar et al., 2022; EFSA, 2012). In their study, Cegar et al. (2022) proposed satisfactory, acceptable or unsatisfactory values for PHC for *Enterobacteriaceae*, which were 1 log_10_ higher than the existing values for carcasses of pigs, in order to address inherently “dirtier” chicken slaughter and dressing process (Buncic et al., 2017). These proposed values were m = 4 log_10_ CFU/g and M = 5 log_10_ CFU/g, where values over ten sampling sessions (usually a 10-week period) giving a result of ≤ m were deemed satisfactory, values between m and M were considered acceptable, and if values were > M the results for that abattoir was unsatisfactory indicating the needs for improvements in hygiene and process controls.

Abattoir interventions are commonly used at slaughter and some of them have been proven very effective in reducing microbiological contamination (Antic et al., 2021; Zdolec et al., 2022). A substantial number of physical interventions to reduce the prevalence and numbers of *Campylobacter* on carcasses during slaughter, such as inside-outside carcass wash, ultrasound combined with steam, rapid carcass surface cooling, etc, have been used in poultry industry in recent times, with more or less success (Gichure et al., 2022). These interventions aim to ensure that the FBOs succeed in meeting PHC and produce carcasses with very low *Campylobacter* load (Buncic et al., 2017).

This study was conducted to evaluate the impact of key abattoir process steps on the levels of *Campylobacter* spp. and *Enterobacteriaceae* on chicken carcasses, including decontaminating effect of an innovative non-chemical abattoir intervention combining hot water carcass immersion and ultrasound with final forced dry air chilling. Furthermore, antimicrobial susceptibility of *Campylobacter* spp. isolates was investigated for antibiotics prioritized as key targets of stewardship programs and monitoring (WHO, 2019a).

## Materials and methods

### Abattoir

Sampling was performed in one commercial abattoir for broilers in England, in the period of 7 weeks from October to December 2021. Abattoir was processing two different sizes of broilers, from 1.5 to 2.5 kg (age range between 28 and 41 days), at different time of day. This reflected on the line speed (140 birds per minute (bpm) for smaller and 110 bpm for larger birds), with a total average number of around 70,000 birds a day. “Soft scalding” method was used, with lower water temperatures of 49°C, scalding time of 4 min and water changed once a day. All process steps were automated and post-evisceration pressure inside-outside wash was used for 7 s with water pressure of 6 bar. After this wash, carcasses were immersed in a hot water at 72°C for 5 s, raising the temperature on their surface to around 50°C (to ensure no organoleptic damages to their skin), while being exposed to ultrasound treatment (the frequency was confidential to the manufacturer). The treatment has its own integrated water recirculation unit, remote monitoring and a limp home mode in the event of breakdown. Carcasses are immersed in a “pocket” of water during the treatment which prevents back contamination and airborne dispersal of the bacteria. The ultrasound generates vacuum bubbles which deliver microseconds of very intense heat on implosion, damage the cell membrane and force the bacteria off the surface of the carcass. This “cavitation” on the surface of the chicken carcass also enhances the energy transfer from the process tank water to the carcass surface and bacteria. This contaminated water is then transferred to another tank for cleaning and recirculation. After the intervention, carcasses were going through an integrated spraying with cold water for 5 s to reduce their surface temperatures. Finally, carcasses were air-chilled using forced dry air system at 0°C for 75–85 min before they were graded and packed.

### Sampling of carcasses

Seven batches of broilers slaughtered on seven processing days were sampled. From each batch, 10 neck skin samples were taken aseptically and at random. New sterile gloves were used for each sampling point to prevent cross-contamination. Neck skin was collected by turning the zip-lock bag inside out over the hand, gripping the neck skin with the hand covered by the bag, and then cutting large portion of it using sterile scissors for each sample (amounting to approximately 30 g). Samples were taken immediately after four process steps: Defeathering, evisceration, hot water immersion/ultrasound intervention and air-chilling (40 samples per day/batch). Post-defeathering sampling point was set as a baseline to evaluate the impact of other key abattoir process steps on microbial loads (evisceration, intervention and chilling). No post-intervention samples were taken for batch 5 because on that day, the intervention was not operational. Therefore, a total of 270 neck skin samples were collected. Detailed sampling plan is provided in Table 1. Samples were transported to a laboratory within 2 h in the cooling box. Food chain information for each batch were also collected to analyze birds’ health problems and antimicrobial usage on farms.

**TABLE 1:** Sampling plan outlining the processing steps after which the neck skin samples were collected, number of samples collected in each batch and order of processing in the abattoir on the sampling day.

Sampling point	Batch and order of processing on the sampling day							Total
	1 (4th)	2 (last)	3 (last)	4 (1st)	5 (4th)	6 (3rd)	7 (2nd)	
Post-defeathering	10	10	10	10	10	10	10	70
Post-evisceration	10	10	10	10	10	10	10	70
Post-intervention	10	10	10	10	0	10	10	60
Post-chilling	10	10	10	10	10	10	10	70
Total	40	40	40	40	30	40	40	270

### Quantification of *Campylobacter* spp. and *Enterobacteriaceae*

Samples were weighed to 25 g and suspended in 25 mL of Maximum Recovery Diluent (MRD) to create undiluted 1:1 sample (10°). This suspension was homogenized in the stomacher for 1 min and left for 15 min to allow time for the sample to settle and microorganisms to resuscitate. To make the 10^1^ dilution, 1 mL of the undiluted sample was added to 9 mL of MRD. This dilution process was repeated until the necessary dilutions were reached. For quantification of *Enterobacteriaceae* the inoculum was plated onto *Enterobacteriaceae* count Petrifilms (3M Health Care) and incubated at 37°C for 24 h (NF Validation Certificate Number 3M 01/6–09/97, as validated against ISO 21528 part 2 VRBG method). For quantification of *Campylobacter*, the inoculum was plated onto Modified Charcoal Cefperazone Deoxycholate Agar (mCCDA) supplemented with cefoperazone (32 mg/l) and amphotericin B (10 mg/l) (Oxoid, United Kingdom) and incubated at 41.5°C in a microaerophilic environment for 44 h, based on ISO 10272–2:2017 (Jacobs-Reitsma et al., 2019).

### *Campylobacter* spp. confirmation

Following incubation, up to four single colonies of different morphology were picked from mCCDA and were then plated onto Columbia blood agar containing 5% (v/v) defibrinated horse blood. Plates were incubated for 48 h under both microaerophilic environment at 41.5°C and aerobic environment at 30°C, to distinguish morphologically similar *Campylobacter* and *Aliarcobacter*, (formerly *Arcobacter*) species. Up to four single colonies of different morphology were stored at –80°C in Microbank vials (Pro-Lab, Bromborough, United Kingdom. Cultures incubated under microaerophilic environment were then analyzed on the Bruker MALDI-TOFF Biotyper (Bruker Daltonics GmbH & Co. KG.) with the aim to identify the isolates at species level. Formic acid was added when setting up the plates for the MALDI-TOFF analysis to ensure that the wall of Gram-positive cells was broken if present. Where MALDI-TOFF did not confirm them as *Campylobacter* genus, PCR was performed to allow further identification. For this, chromosomal DNA was extracted by mixing 20 μL of the frozen bacterial stock with 300 μL of Chelex solution (Chelex-100, Bio-Rad) and heated at 95°C for 10 min. The mixture was then centrifuged (16,200 g for 3 min) and 50 μL of the supernatant collected and mixed with 450 μL of sterilized distilled water. A multiplex PCR based on differences in the lpxA gene to determine the *Campylobacter* species (Klena et al., 2004) was performed and when negative, it was followed by a multiplex PCR based on the 16S rRNA gene sequences for the identification of the genera *Campylobacter* spp. (Katzav et al., 2008; Linton et al., 1996) and *Aliarcobacter* spp. (González et al., 2000).

### Antimicrobial susceptibility testing of *Campylobacter* spp.

Antimicrobial susceptibility of *Campylobacter* spp. isolates was evaluated via disc diffusion method as per European Committee on Antimicrobial Susceptibility Testing (EUCAST) 2020 guidelines on Mueller-Hinton + 5% mechanically defibrinated horse blood + 20 mg/L β-NAD Plates. A sterile loop was used to transfer cultures from the Columbia culture media into 3 mL of saline solution until the suspension was to 0.5 McFarland standard. A swab was then used to create a uniform “bacterial rug” on Mueller Hinton Agar with 5% Sheep Blood agar. Antibiotic discs (MAST Group Ltd.) for aminoglycosides (gentamicin 10 and streptomycin 300 μg), quinolones (ciprofloxacin 5 μg and nalidixic acid 30 μg), macrolides (erythromycin 15 μg), and tetracyclines (30 μg) were applied using a dispenser and plates were incubated at 41.5°C under microaerophilic conditions for 48 h. Each antibiotic disk’s zone of inhibition (ZOI) was measured in millimeters. For most antibiotics the EUCAST human clinical breakpoints were used to categorize response as: Susceptible (S) or resistant (R). When the zone of no growth was in between the clinical breakpoints for resistance or susceptibility, the category of “susceptible, increased exposure” (I) was used. For the antibiotics for which these didn’t exist, EUCAST 2016 Epidemiological Cut off (ECOFF), British society for Antimicrobial Chemotherapy (BSAC) and Clinical and Laboratory Standards Institute (CLSI) standards were used instead. This applied, respectively, to gentamicin, nalidixic acid and streptomycin. In relation to the CLSI standards for streptomycin sensitivity, there was none available that were specific to *Campylobacter* so the standards for *Enterobacteriaceae* family were used instead. Table 2 summarizes the breakpoints used for data interpretation.

**TABLE 2:** Source of standards and breakpoints used for antibiotic sensitivity disc diffusion testing.

Antibiotic	Standard	Zone diameter breakpoints (mm)			
		R < (*C. jejuni*)	S > (*C. jejuni*)	R < *(C. coli*)	S > = (*C. coli*)
Ciprofloxacin	EUCAST 2023	26	50		
Erythromycin	EUCAST 2023	20	20	24	24
Gentamicin	EUCAST 2016 ECOFF	20	20		
Nalidixic acid	BSAC *vs.* 14 (2015)	19	20		
Streptomycin	CSLI M100 (2016)	11	15		
Tetracycline	EUCAST 2023	30	30		

R, resistant; S, sensitive; EUCAST, European Committee on Antimicrobial Susceptibility Testing; ECOFF, Epidemiological Cut Off; BSAC, British society for Antimicrobial Chemotherapy; CLSI, Clinical and Laboratory Standards Institute.

### Data analysis

Microbial counts obtained from neck skin samples were transformed to log_10_ CFU/g values for subsequent data analysis. The detection limit was 1 CFU/g of neck skin. Bacterial load was compared between sampling points within the same batch and as a mean log_10_ between sampling points. Mean logs between sampling days and sampling points were compared using a *t*-test (two-tailed, assuming equal variances) in Excel at the significance level of *p* ≤ 0.05. To adjust for multiple comparisons and reduce the risk of Type I errors, Bonferroni correction was applied at a significance level set at 0.05.

## Results

### Impact of key abattoir process steps on carcass microbial contamination

All 270 samples cultured on mCCDA produced presumptive *Campylobacter* spp. colonies, but MALDI-TOFF and PCR confirmed 261 *Campylobacter* spp. (96.7%), of which 253 were identified as *C. jejuni* (93.7% of the total number of samples), three were identified as *C. coli* (1.1%) and five samples were identified as *Campylobacter* spp. (1.9%). Further three isolates were found to belong to *Aliarcobacter* genera and six were unidentifiable (3.3% in total non-*Campylobacter* samples). Only samples where *Campylobacter* presence was confirmed were included in further quantitative analyses of process hygiene in this abattoir.

**FIGURE 1:**
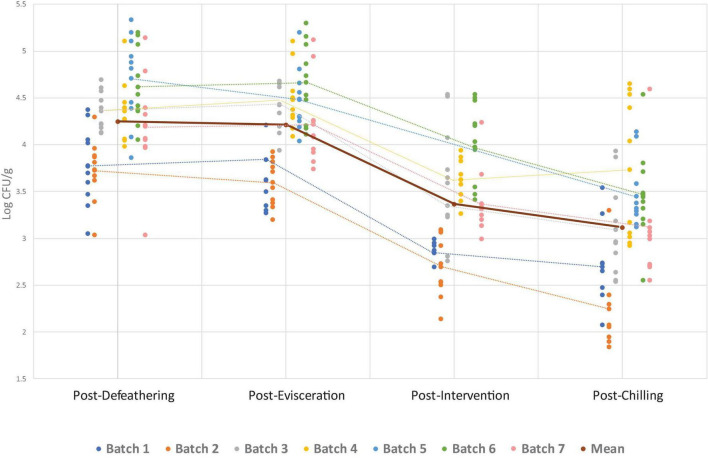
*Campylobacter* counts per batch (processing day) at separate sampling points. Individual data points show counts for each batch, while dotted lines connect the mean count within each batch at each sampling point. The solid line represents the overall mean across all batches at each sampling point. For batch 5, the intervention was not in operation on the sampling day, so no counts are available for this point.

**FIGURE 2:**
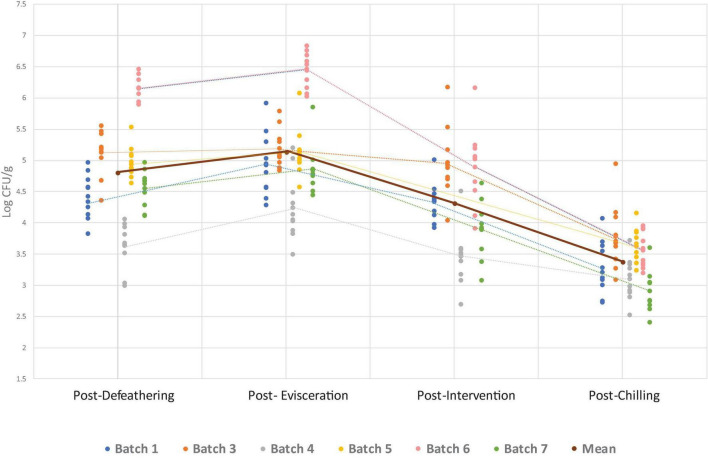
*Enterobacteriaceae* counts per batch (processing day) at separate sampling points. Individual data points show counts for each batch, while dotted lines connect the mean count within each batch at each sampling point. The solid line represents the overall mean across all batches at each sampling point. Note: no data on *Enterobacteriaceae* is available from batch 2 and for batch 5, the intervention was not in operation on the sampling day, so no counts are available for this point.

The overall *Campylobacter* and *Enterobacteriaceae* counts found at four sampling points for all seven batches are presented in Figures 1, 2. The numerical data can be found in Supplementary Table 1. Both *Campylobacter* and *Enterobacteriaceae* initial and final counts were very variable between processing days and decreased on average from 4.3 log_10_ CFU/g to 3.1 log_10_ CFU/g and 4.8 log_10_ CFU/g to 3.3 log_10_ CFU/g, respectively. Mean *Campylobacter* levels on final carcasses after chilling varied between seven batches from 2.2 log_10_ CFU/g to 3.7 log_10_ CFU/g, and for *Enterobacteriaceae* between six sampled batches from 2.9 log_10_ CFU/g to 3.8 log_10_ CFU/g. If these results were taken into the context of compliance with the regulatory PHC for *Campylobacter*, a satisfactory result for the abattoir, using PHC limits applicable at the time of the sampling conducted in this study, would be obtained if 15/50 samples (or 30%) during the sampling window (10 weeks) did not exceed 1,000 CFU/g (3 log_10_) in chicken neck skins after chilling. In our study, 70 samples were obtained in total after chilling during 7 weeks of sampling, in which 40 samples were found exceeding 1,000 CFU/g of *Campylobacter* (57.1%), ranging from 10 to 100% in individual weeks (Table 3). The interpretation of PHC for this abattoir regarding compliance with *Campylobacter* levels was unsatisfactory overall in the sampling window, with only two initial weeks of sampling giving satisfactory result. Regarding *Enterobacteriaceae* counts, 60 samples were obtained after chilling during 6 weeks of sampling and all daily mean results were under 4 log_10_ CFU/g giving satisfactory result in all weeks, based on the limits proposed by Cegar et al. (2022) and Hauge et al. (2023) (Table 3).

**TABLE 3:** *Campylobacter* and *Enterobacteriaceae* counts from neck skin after chilling for the purpose of process hygiene criteria evaluation.

Batch	*Campylobacter*^a^ (number of samples with > 1,000 CFU/g)	Interpretation of the results	*Enterobacteriaceae*^b^ (daily mean log_10_ CFU/gr)	Interpretation of the results
1	2/10 (20%)	Satisfactory	3.3	Satisfactory
2	1/10 (10%)	Satisfactory	ND	/
3	4/10 (40%)	Unsatisfactory	3.8	Satisfactory
4	8/10 (80%)	Unsatisfactory	3.1	Satisfactory
5	10/10 (100%)	Unsatisfactory	3.6	Satisfactory
6	9/10 (90%)	Unsatisfactory	3.6	Satisfactory
7	6/10 (60%)	Unsatisfactory	2.9	Satisfactory
Total	40/70 (57.1%)	Unsatisfactory	3.3	Satisfactory

^a^PHC limits set to values applicable at the time of the sampling conducted in this study, if 15/50 samples (or 30%) did not exceed 1,000 CFU/g (3 log_10_), then the result is satisfactory; from 1.1.2025. limits changed to no more than 10/50 samples (or 20%) for a satisfactory result.

^b^PHC limits arbitrarily set at m = 4 log_10_ CFU/g, M = 5 log_10_ CFU/g; a result of ≤ m is satisfactory, between m and M acceptable, and > M is unsatisfactory. ND, no data for respective day.

Microbial levels pre- and post- the key process steps, evisceration, intervention and chilling, are presented in Table 4. On average, *Campylobacter* levels did not change between defeathering and evisceration (from 0.12 log_10_ increase to 0.23 log_10_ decrease between batches), while levels of *Enterobacteriaceae* increased by 0.33 logs, but not significantly (0.07– 0.61 log_10_ between batches). Hot water immersion and ultrasound intervention substantially decreased *Campylobacter* levels by 0.85 log_10_ and *Enterobacteriaceae* levels similarly by 0.82 log_10_, but with no statistical significance (0.63–0.9 log_10_ and 0.23 log_10_–1.55 log_10_, respectively). Forced dry air chilling was highly effective in reducing *Enterobacteriaceae* levels by 0.93 log_10_ but had very variable effect across all batches and overall insignificant reduction effect on *Campylobacter* of 0.25 log_10_ (from 0.11 log_10_ increase to 1.03 log_10_ decrease). Nevertheless, overall impact of all key process steps on final carcass microbial load, from the baseline point of defeathering to the final chilled carcass ready for packaging, resulted in overall significant reduction in *Campylobacter* of 1.14 log_10_ (*p* < 0.01) and high but not significant reduction in *Enterobacteriaceae* levels of 1.43 log_10_. The reductions were significant in all but one batch, from 0.63 log_10_ to 1.48 log_10_ in case of *Campylobacter*, and from 0.53 to 2.59 log_10_ for *Enterobacteriaceae*.

**TABLE 4:** Log change in *Campylobacter* and *Enterobacteriaceae* counts after selected abattoir process steps.

		*Campylobacter*		*Enterobacteriaceae*	
Batch	Process steps	Log_10_ change	*P*-value	Log_10_ change	*P*-value
1	Defeathering—evisceration	–0.15	0.41	0.50	0.02
	Evisceration—intervention	–0.74	< 0.01*	–0.59	0.006
	Intervention—chilling	–0.15	0.47	–1.05	< 0.01*
	Overall change (defeathering—chilling)	–1.04	< 0.01*	–1.11	< 0.01*
2	Defeathering—evisceration	–0.13	0.34	ND	ND
	Evisceration—intervention	–0.90	< 0.01*	ND	ND
	Intervention—chilling	–0.45	0.022	ND	ND
	Overall change (defeathering—chilling)	–1.48	< 0.01*	ND	ND
3	Defeathering—evisceration	0.06	0.56	0.07	0.64
	Evisceration—intervention	–0.84	< 0.01*	–0.23	0.28
	Intervention—chilling	–0.50	0.07	–1.19	< 0.01*
	Overall change (defeathering—chilling)	–1.27	< 0.01*	–1.34	< 0.01*
4	Defeathering—evisceration	0.12	0.44	0.61	0.008
	Evisceration—intervention	–0.85	< 0.01*	–0.79	0.002
	Intervention—chilling	0.11	0.68	–0.36	0.06
	Overall change (defeathering—chilling)	–0.63	0.029	–0.53	0.003
5^a^	Defeathering—evisceration	–0.23	0.24	0.15	0.31
	Evisceration—chilling	–1.03	< 0.01*	–1.51	< 0.01*
	Overall change (defeathering—chilling)	–1.26	< 0.01*	–1.35	< 0.01*
6	Defeathering—evisceration	0.05	0.80	0.29	0.015
	Evisceration—intervention	–0.63	0.004	–1.55	< 0.01*
	Intervention—chilling	–0.57	0.015	–1.33	< 0.01*
	Overall change (defeathering—chilling)	–1.15	< 0.01*	–2.59	< 0.01*
7	Defeathering—evisceration	0.02	0.93	0.29	0.08
	Evisceration—intervention	–0.85	< 0.01*	–0.95	< 0.01*
	Intervention—chilling	–0.29	0.19	–0.98	< 0.01*
	Overall change (defeathering—chilling)	–1.13	< 0.01*	–1.64	< 0.01*
Average	Defeathering—evisceration	–0.04	0.87	0.33	0.49
	Evisceration—intervention	–0.85	0.007	–0.82	0.08
	Intervention—chilling	–0.25	0.40	–0.93	0.013
	Overall change (defeathering—chilling)	–1.14	< 0.01*	–1.43	0.003

*P*-values were compared to a Bonferroni-corrected alpha level (α = 0.05/number of comparisons), which was calculated at α≈0.00192 (26 comparisons) for *Campylobacter* and α≈0.00208 (24 comparisons) for *Enterobacteriaceae*.; *indicate a statistically significant increase or decrease in CFU/g; ***^a^***intervention was not operational on the sampling day; ND, no data for respective batch.

### Antibiotic resistance profiles of *Campylobacter* spp.

In terms of overall resistance to antibiotics when all batches were pooled together, 48.7% of isolates (124 out of 255) showed resistance to at least one antibiotic (Table 5) and 12% of isolates (30 out of 255) showed no resistance to any antibiotic. Almost 50% of isolates showed susceptibility with increased exposure to ciprofloxacin and streptomycin (Table 6). Most isolates showed only susceptibility with increased exposure to ciprofloxacin (49%), and exhibited clear resistance to tetracycline (45%), nalidixic acid (41%), and ciprofloxacin (39%). Only 9/255 *Campylobacter* isolates showed resistance to erythromycin, streptomycin and gentamicin (2, 1, and 1%, respectively).

**TABLE 5:** Antibiotic resistance prevalence in *Campylobacter* isolates from chicken neck skin.

Susceptibility status										
	Resistant						Susceptible, increased exposure		Susceptible	
AB Class	Quinolones		Aminoglycosides		Tetracyclines	Macrolides	Quinolones	Amino-glycosides		
AB	CPX	NA	G	S	T	E	CPX	S		
Batch 1	28% (7/25)	48% (12/25)	4% (1/25)	0% (0/25)	84% (21/25)	12% (3/25)	72% (18/25)	0% (0/25)	0% (0/25)	91–100%
Batch 2	95% (37/39)*	97% (38/39)	0% (0/39)	0% (0/39)	97% (38/39)	5% (2/39)	3% (1/39)	0% (0/39)	0% (0/39)	81–90%
Batch 3	13% (5/40)*	13% (5/40)	0% (0/40)	0% (0/40)	15% (6/40)	0% (0/40)	88% (35/40)	0% (0/40)	3% (1/40)	71–80%
Batch 4	8% (3/40)	3% (1/40)	0% (0/40)	0% (0/40)	0% (0/40)	0% (0/40)	58% (23/40)	0% (0/40)	35% (14/40)	61–70%
Batch 5	0% (0/30)	0% (0/30)	0% (0/30)	0% (0/30)	0% (0/30)	0% (0/30)	63% (19/30)	0% (0/30)	37% (11/30)	51–60%
Batch 6	88% (35/40)*	88% (35/40)	3% (1/40)	3% (1/40)	98% (39/40)	0% (0/40)	13% (5/40)	0% (0/40)	0% (0/40)	41–50%
Batch 7	29% (12/41)*	32% (13/41)	0% (0/41)	2% (1/41)	27% (11/41)	0% (0/41)	61% (25/41)	2% (1/41)	10% (4/41)	31–40%
All batches	39% (99/255)	41% (104/255)	1% (2/255)	1% (2/255)	45% (115/255)	2% (5/255)	49% (126/255)	1% (1/255)	12% (30/255)	21–30%
										11–20%
										1–10%
										0–0.99%

CPX, ciprofloxacin; NA, nalidixic acid; G, gentamycin; S, streptomycin; T, tetracycline; E, erythromycin; Percentages have been rounded to the nearest whole number. *In these batches all isolates that showed resistance to ciprofloxacin also showed resistance to nalidixic acid.

**TABLE 6:** Antibiotic resistance profiles in *Campylobacter* isolates from chicken neck skin.

Resistance profile	Overall% (*n* = 255)	% Per batch							
		Batch 1 (*n* = 25)	Batch 2 (*n* = 39)	Batch 3 (*n* = 40)	Batch 4 (*n* = 40)	Batch 5 (*n* = 30)	Batch 6 (*n* = 40)	Batch 7 (*n* = 41)	
CPX	1.2% (*n* = 3)	–	–	–	7.5% (*n* = 3)	–	–	–	91–100%
CPX, NA, S	0.4% (*n* = 1)	–	–	–	–	–	–	2.4% (*n* = 1)	81–90%
CPX, NA, T	34.5% (*n* = 88)	16% (*n* = 4)	89.7% (*n* = 35)	12.5% (*n* = 5)	–	–	82.5% (*n* = 33)	26.8% (*n* = 11)	71–80%
CPX, NA, T, E	1.6% (*n* = 4)	8% (*n* = 2)	5.1% (*n* = 2)	–	–	–	–	–	61–70%
CPX, NA, T, G	0.8% (*n* = 2)	4% (*n* = 1)	–	–	–	–	2.5% (*n* = 1)	–	51–60%
CPX, NA, T, S	0.4% (*n* = 1)	–	–	–	–	–	2.5% (*n* = 1)	–	41–50%
E, NA	0.4% (*n* = 1)	4% (*n* = 1)	–	–	–	–	–	–	31–40%
N A	1.6% (*n* = 4)	4% (*n* = 1)	2.6% (*n* = 1)	–	2.5% (*n* = 1)	–	–	2.4% (*n* = 1)	21–30%
NA, T	1.2% (*n* = 3)	12% (*n* = 3)	–	–	–	–	–	–	11–20%
T	6.7% (*n* = 17)	44% (*n* = 11)	2.6% (*n* = 1)	2.5% (*n* = 1)	–	–	10% (*n* = 4)	–	1–10%
Total	48.7% (*n* = 124)	92% (*n* = 23)	100% (*n* = 39)	15% (*n* = 6)	10% (*n* = 4	0%	97.5% (*n* = 39)	31.7% (*n* = 13)	0–0.99%

The percentage for overall represents results from all batches pooled together; the data related to individual batches represent the percentage within the batch. CPX, ciprofloxacin; NA, nalidixic acid; G, gentamycin; S, streptomycin; T, tetracycline; E, erythromycin; n, number of isolates. Percentages have been rounded to the nearest decimal.

Ten different resistance profiles were identified, with the resistance to the quinolones and tetracycline most commonly shared (34.5%) (Table 5). Multidrug resistance (resistance to at least one antibiotic in three or more different classes) was found in seven isolates, to quinolones in combination with resistance to gentamycin (0.8%), erythromycin (1.6%) or streptomycin (0.4%). To note, all isolates identified in batches 2, 3, 6, and 7 that exhibited resistance to ciprofloxacin also exhibited resistance to nalidixic acid.

Antibiotic resistance prevalence in seven batches ranged from 0 to 100% and was highest in batches 2, 6, and 1 (100, 97.5, and 92%, respectively, Table 5). While all chicken batches met drug withdrawal period requirements, food chain information indicated prior antimicrobial exposure in batch 1 and batch 2. Batch 1 had a history of enteritis, wet litter and lameness, and birds received Amoxicillin trihydrate at 6–10 days and 24–26 days; and Doxycycline hyclate, at 10–13 days. Isolates from batch 1 demonstrated widespread antibiotic resistance, with no isolate found sensitive to all six tested antibiotics and two isolates showing susceptibility with increased exposure to ciprofloxacin (8%). Predominant resistances were found to tetracycline (84%), ciprofloxacin (28% resistance, 72% increased exposure) and nalidixic acid (48%), and less to erythromycin (12%) and gentamycin (4%). Multidrug resistance was found in three isolates resistant to quinolones and tetracycline, combined with erythromycin and gentamycin (Table 6). Batch 2 had a history of yolk sac infection and small bird culling, and birds received Amoxicillin trihydrate at 4–6 days and Doxycycline hyclate at 6–11 days. Similarly to batch 1, all *Campylobacter* isolates in batch 2 exhibited resistance to at least one antibiotic. Most prevalent was resistance to tetracycline and nalidixic acid (97% each), ciprofloxacin (95% resistance, 3% increased exposure) and with much lower prevalence to erythromycin (5%). Multidrug resistance was found in two isolates resistant to quinolones, tetracycline and erythromycin. Isolates from batch 3 and batch 4 exhibited limited antibiotic resistance and mostly showed increased exposure to ciprofloxacin (88 and 58% respectively). *Campylobacter* isolates from batch 5 showed minimal resistance, with 37% being sensitive to all antibiotics tested and mostly exhibiting increased exposure to ciprofloxacin (63%). On the other hand, almost all isolates from batch 6 showed some resistance to antibiotics, with highest resistance found to tetracycline (98%), ciprofloxacin (88% resistance, 13% increased exposure) and nalidixic acid (88%). Two multidrug resistant isolates were found exhibiting resistance to quinolones, tetracycline and aminoglycosides. Finally, more than two third of isolates in batch 7 showed some resistance or increased exposure to antibiotics, with highest resistance found to nalidixic acid (32%), ciprofloxacin (29% resistance, 61% increased exposure) and tetracycline (27%).

## Discussion

This study demonstrated that the abattoir processing substantially decreased *Campylobacter* spp. and *Enterobacteriaceae* levels, from the baseline point of defeathering to the final chilled carcass ready for packaging, by 1.14 log_10_ and 1.43 log_10_, respectively. The major contributing step in this was intervention based on the combined effect of hot water immersion and ultrasound, which decreased *Campylobacter* levels by 0.85 log_10_ and *Enterobacteriaceae* levels similarly by 0.82 log_10_ (but with no statistical significance). Forced dry air chilling was effective in reducing *Enterobacteriaceae* levels by 0.93 log_10_ (*p* < 0.05), but ineffective in reducing *Campylobacter* (0.25 log_10_, *p* > 0.05). Evisceration expectedly did not have major effects on contamination, with levels of *Enterobacteriaceae* only slightly increasing but not significantly by 0.33 logs, indicating that this processing step was properly performed in this abattoir, i.e., machinery calibrated in such a way to avoid gut spillage and contamination. While direct comparisons with other published studies are often difficult due to variable conditions between abattoirs, data available in the literature often show similar trends, with reduction in contamination during scalding, then significant increase during defeathering, usually no change or very small increase in contamination as a consequence of evisceration, and certain reductions achieved during chilling (Buncic et al., 2017). Similar impact of abattoir processing on the levels of *Campylobacter* spp. and *Enterobacteriaceae* on final broiler carcasses was also observed in studies by Hauge et al. (2023) (reductions of 1.0 log_10_ and 1.1 log_10_, respectively) and Emanowicz et al. (2021) (reduction of 0.8 log_10_ for *Campylobacter*). On contrary, reductions of less than 0.5 log_10_ were reported in studies by Zweifel et al. (2015), Althaus et al. (2017), and Roccato et al. (2018) for both *Campylobacter* spp. and *Enterobacteriaceae.* This abattoir used scalding system where the water was changed only once a day, which could imply a possible build-up of contamination during the day and expected increase in microbial load on the carcasses processed last on the day. However, our findings show that some batches processed last on the day (e.g., batches 2 and 3) had lower *Campylobacter* load than those processed earlier in the day (e.g., batches 4, 5, and 6), which indicated no clear carcass contamination pattern. There may have been other contamination sources for the carcasses on the slaughterline, which could have been investigated by sampling the machinery and environment, but this was outside the scope of our study.

One of the aims in this study was to investigate the effectiveness of the intervention implemented in this abattoir and to our knowledge, this is the first study to present data on hot water immersion and ultrasound intervention for chicken carcasses. Two other similar studies (Moazzami et al., 2021; Musavian et al., 2014) investigated effect of ultrasound combined with hot steam, the SonoSteam system, where steam was used at 90–94°C and ultrasound at 30–40 kHz for 15–20 min, with pooled effect of 1.25 log_10_ reduction in *Campylobacter* found in meta-analysis conducted on five trials presented in those two studies (Gichure et al., 2022). In a more recent study, Musavian et al. (2022) found 0.8 log_10_ reduction for *Campylobacter* in neck skin samples achieved with the SonoSteam. Reported reduction effects on *Enterobacteriaceae* with the SonoSteam were similar to *Campylobacter* in three published studies and were 0.6 log_10_ (Moazzami et al., 2021) and 1.1 log_10_ (Musavian et al., 2022). All these reported reductions are in line with our study, and the intervention usually has a little to no effect on the organoleptic features on the final product (Lauteri et al., 2023; Musavian et al., 2014). This strongly suggests that this physical intervention can be recommended for use in chicken abattoirs. Potable water-based interventions that do not use chemicals are considered acceptable by the industry and consumers, as their implementation does not require specific approval by the regulatory authorities (Antic et al., 2021). Indeed, the EU Regulation 853/2004 specifies that the FBOs must not use any substance other than potable water to remove surface contamination from products of animal origin and allows, in principle, the use of other decontamination treatments following appropriate consideration and a risk assessment by EFSA and approval by the regulatory authorities (EC, 2004).

The initial reason for implementing the hot water immersion and ultrasound intervention in this abattoir was to contribute to overall reduction of *Campylobacter* levels on final chicken carcasses, enabling this FBO to meet the PHC. Testing within the PHC framework is intended as an objective verification measure in the HACCP system allowing abattoirs to take some actions and improve process hygiene. Indirectly, the results of this testing can also be used to communicate the level of *Campylobacter* risk to consumers purchasing chicken meat processed in abattoirs, in the current absence of food safety criteria for *Campylobacter*. Even though this intervention achieved significant reduction effects on *Campylobacter*, the final *Campylobacter* levels on carcasses post-chilling during the sampling window of 7 weeks exceeded the regulatory limits, indicating that the level of hygiene in this abattoir during this period was unsatisfactory based on the current PHC. Very high levels of *Campylobacter* were observed on the final carcasses in some weeks, e.g., in weeks 4, 5, and 6, from 3.5 log_10_ CFU/g to 3.7 log_10_ CFU/g. This, alongside with the observed higher initial *Campylobacter* levels post-defeathering (likely reflecting on-farm status), suggests that the reduction capacity of the hot water immersion and ultrasound intervention was possibly exceeded. No single intervention has been shown to be capable of eliminating *Campylobacter* and a “multiple hurdle” approach would be necessary to produce carcasses with low *Campylobacter* levels and consequently reduce campylobacteriosis cases in humans (ACMSF, 2019; Goddard et al., 2022). Nevertheless, if *Enterobacteriaceae* counts were used as indicator microorganism in PHC testing, this abattoir would have achieved a satisfactory result and met PHC based on the limits proposed by Cegar et al. (2022) and Hauge et al. (2023). These indicators are suited for PHC as they indicate fecal contamination and are always present in the process. During the sampling window, contamination with both *Campylobacter* and *Enterobacteriaceae* was gradually increasing and peaking in weeks 4–6 after which contamination started decreasing in week 7 when our study finished. This fluctuation in contamination is expected and the FBO must ensure to conduct the trend analysis of the data, and root cause analysis to investigate the problem and rectify it as soon as possible. As Cegar et al. (2022) and Hauge et al. (2023) noted, testing for both *Campylobacter* and *Enterobacteriaceae* can be used to risk categorize abattoirs and compare the performances between different abattoirs. In our study, this abattoir would likely be categorized as low-risk based on the *Enterobacteriaceae* results, and medium-risk based on (mostly borderline) *Campylobacter* results. However, based on our observations and impression, it is more likely that in the long run, proactive attitude of abattoir’s management in implementing innovative abattoir intervention and overall good slaughter hygiene, would place this abattoir under low-risk category.

Antimicrobial susceptibility of *Campylobacter* spp. isolates was also investigated in this study for antibiotics prioritized as key targets of stewardship programs and monitoring. *Campylobacter* isolates were tested for resistance against aminoglycosides (gentamicin and streptomycin), macrolides (erythromycin), quinolones (ciprofloxacin and nalidixic acid) and tetracycline, among which three of these are in the WHO AWaRe Classification Database Watch group (ciprofloxacin, streptomycin, erythromycin) and two in the Access group (gentamycin, tetracycline) (WHO, 2019a). These antimicrobials are either critically important (macrolides, quinolones, aminoglycosides) or highly important for public health (tetracycline) (WHO, 2019b). As expected, high levels of resistance to ciprofloxacin (39%) and tetracycline (45%) were found. This was broadly in line with previous UK surveys of retail chicken meat between 2015 and 2020 where ciprofloxacin resistance in *C. jejuni* ranged between 41 and 54% and tetracycline resistance between 52 and 68% (Fearnley and Rodgers, 2023; Jorgensen et al., 2021). In the most recent UK study by Fearnley and Rodgers (2023), ciprofloxacin resistance was detected in 65.4% of *C. jejuni* isolates and tetracycline resistance in 73.8% in chicken meat retail samples, which is higher than in our study. These authors noted that the prevalence of resistance to these antibiotics is not declining in *C. jejuni* on poultry meat, despite the successful efforts of the British Poultry Council to antimicrobial stewardship scheme to reduce the antimicrobial use in poultry meat chain (Fearnley and Rodgers, 2023; UKVARSS, 2022). Resistance to erythromycin, streptomycin and gentamicin was very low (2, 1, and 1%, respectively), which was also in line with previous UK studies (Fearnley and Rodgers, 2023; Jorgensen et al., 2021). Multidrug resistance, which is characterized as the acquired ability to be resistant to at least one antimicrobial agent in three or more different classes (Magiorakos et al., 2012), was found in only 2.7% of *Campylobacter* isolates. However, there were four multidrug resistant *Campylobacter* isolates that were co-resistant to both ciprofloxacin and erythromycin (plus tetracycline), in 1.6% of samples, that had very high counts of 700 CFU/g, 5,000 CFU/g, 5,500 CFU/g, and 7,500 CFU/g. This is of a potential public health concern because erythromycin is the main treatment option for complex cases of campylobacteriosis and both ciprofloxacin and erythromycin are classified as a highest priority critically important antimicrobial (HP-CIA) by the WHO (WHO, 2019b).

All batches except for batch 5 exhibited clear antimicrobial resistance to at least one antibiotic with 48.7% *Campylobacter* isolates found to be resistant overall. Batches 1, 2, and 6 had very high resistance to tetracycline of 84, 97, and 98%, respectively. Of these, batch 1 and batch 2 had both a history of disease and antibiotic treatment with Doxycycline hyclate, a tetracycline antibiotic. This study was also a part of a wider research of antimicrobial resistance in broiler meat production chain by Abdulla (2024), where the same neck skin samples were analyzed for antimicrobial resistant and extended-spectrum beta-lactamase (ESBL)-producing *E. coli*. The author found that 57.4% of the *E. coli* isolates and 25% of ESBL-producing *E. coli* isolates were also resistant to tetracyclines, and 22.2% of *E. coli* isolates and 100% of ESBL-producing *E. coli* isolates were resistant to ciprofloxacin (Abdulla, 2024). Given their broad spectrum nature and affordability, tetracyclines are the second most often used class of antibiotics in the British poultry sector (UKVARSS, 2022). Tetracyclines are considered highly important for public health (WHO, 2019b) and should be used with prudence (European Medicines Agency, 2019).

As a result of the importance of *Campylobacter* in the disease burden, many countries have adopted national surveillance and control systems that have not always been proved fully successful. For example, in the United Kingdom, the Food Standards Agency (FSA) began a *Campylobacter* reduction program in 2010 in collaboration with the industry. The overall goal was to reduce the proportion of chicken meat highly contaminated with *Campylobacter* at retail (> 1,000 CFU/g), which would result in significant reduction (15–30%) in the number of human cases as shown by modeling (FSA, 2010, 2015). A recent study compared European *Campylobacter* surveillance programs and found that 12 European countries, with the exception of France, demonstrated a decline in human reported *Campylobacter* cases since the PHC for *Campylobacter* was implemented in 2018 (Olsen et al., 2024). However, this is not the case in the UK where human cases have remained stable at around 100 per 100,000 population between 2014 and 2019 (UKHSA, 2024), even though the proportion of chicken meat samples at retail with greater than 1,000 CFU/g of *Campylobacter* dropped from 19 to 5% (and have remained stable since then) (Kintz et al., 2024). To investigate the continued high incidence in human cases, the UK FSA commissioned an expert elicitation exercise to generate testable hypotheses that might explain why the reduction in highly contaminated chicken carcasses available at retail did not correspond to decreased levels of human disease (Kintz et al., 2024). In total, 25 hypotheses were generated, but the prevailing opinion was that the reasons were likely complex and multifactorial. Notable reasons were that the *Campylobacter* infectious dose could be lower than assumed 1,000 CFU, which can also be linked to increased chances of infection in the UK aging and vulnerable population. There is also a possibility of an increase in the presence of *Campylobacter* virulent strains in the food as a consequence of selective pressure from interventions used in the food chain. Insufficient and/or inadequate controls on farm and during slaughter and processing may select more resistant strains that may be more virulent for humans, offsetting the reductions achieved in *Campylobacter* prevalence in chicken meat at retail (Kintz et al., 2024). In our study, we did not investigate the virulence characteristics of *Campylobacter* isolates, but we found that 2.7% of *Campylobacter* isolates were associated with multidrug resistance, among which four isolates co-resistant to ciprofloxacin, erythromycin and tetracycline, in samples with very high *Campylobacter* counts of up to 7,500 CFU/g. The presence of multidrug resistant strains alongside high number of *Campylobacter* counts on final carcasses may be of a potential public health concern.

## Data Availability

The raw data supporting the conclusions of this article will be made available by the authors, without undue reservation.
