# Supplementary material for: An evaluation of the impact of abattoir processing on the levels of Campylobacter spp. and Enterobacteriaceae on broiler carcasses
Source: Front Microbiol. 2025 Jul 16;16:1613058. doi: 10.3389/fmicb.2025.1613058 (PMC12307395; doi:10.3389/fmicb.2025.1613058)
Supplement: Supplementary file 1 [file Table_1.docx]

Supplementary Material

# Supplementary Figures and Tables

Table suppl_1. *Campylobacter* and *Enterobacteriaceae* mean counts per batch (processing day) and sampling point.

| Sampling point | Batch | | | | | | | |
| --- | --- | --- | --- | --- | --- | --- | --- | --- |
|  | 1 | 2 | 3 | 4 | 5 | 6 | 7 | Average |
| *Campylobacter* mean log_10_ CFU/gr ± SD¹ | | | | | | | | |
| Post-defeathering | 3.8 ± 0.4 | 3.7 ± 0.3 | 4.4 ± 0.2 | 4.4 ± 0.3 | 4.7 ± 0.5 | 4.6 ± 0.4 | 4.2 ± 0.6 | 4.3 ± 0.4 |
| Post-evisceration | 3.6 ± 0.3 | 3.6 ± 0.3 | 4.4 ± 0.3 | 4.5 ± 0.3 | 4.5 ± 0.3 | 4.7 ± 0.4 | 4.2 ± 0.5 | 4.2 ± 0.4 |
| Post-intervention | 2.8 ± 2.9 | 2.7 ± 0.3 | 3.6 ± 0.6 | 3.6 ± 0.2 | ND* | 4.0 ± 0.4 | 3.4 ± 0.4 | 3.4 ± 0.4 |
| Post-chilling | 2.7 ± 0.5 | 2.2 ± 0.4 | 3.1 ± 0.5 | 3.7 ± 0.8 | 3.5 ± 0.4 | 3.5 ± 0.5 | 3.1 ± 0.6 | 3.1 ± 0.5 |
| *Enterobacteriaceae* mean log_10_ CFU/gr ± SD¹ | | | | | | | | |
| Post-defeathering | 4.4±0.3 | ND | 5.1±0.4 | 3.6±0.4 | 5.0±0.3 | 6.2±0.0 | 4.6±0.3 | 4.8 ± 0.8 |
| Post-evisceration | 4.9±0.5 | ND | 5.2±0.3 | 4.3±0.5 | 5.1±0.4 | 6.5±0.3 | 4.8±0.4 | 5.1 ± 0.7 |
| Post-intervention | 4.3±0.3 | ND | 5.0±0.6 | 3.5±0.5 | ND* | 4.9±0.6 | 3.9±0.5 | 4.3 ± 0.6 |
| Post-chilling | 3.3±0.4 | ND | 3.8±0.5 | 3.1±0.3 | 3.6±0.3 | 3.6±0.3 | 2.9±0.3 | 3.3 ± 0.3 |

**¹** log_10_ CFU/g with standard deviation (SD); ND: no data for respective samples; *The intervention was not in operation on the sampling day.
